# Uneven global distribution of randomized trials in hip fracture surgery

**DOI:** 10.3109/17453674.2012.704562

**Published:** 2012-08-25

**Authors:** Marco Yeung, Mohit Bhandari

**Affiliations:** Division of Orthopaedic Surgery, McMaster University, Hamilton, Ontario, Canada

## Abstract

**Background and purpose:**

Hip fractures are among the top causes of global disability. Conduction of high-quality studies such as randomized controlled trials to assess the effectiveness of interventions remains crucial. The geographic distribution of hip fracture studies is largely unknown. We wanted to make a global assessment of national contributions of randomized controlled trials on surgical interventions for hip fracture.

**Methods:**

We performed a systematic search for randomized controlled trials on surgical interventions for hip fracture that were published from May 1970 to May 2011. Study information including sample size and study location was abstracted. The number of trials and cumulative sample size of hip fracture clinical trials were analyzed with respect to geographic region (city, country, and continent).

**Results:**

We identified 199 randomized trials investigating surgical interventions. Sweden ranked highest with 50 trials (8,941 patients). The United Kingdom followed with 40 trials (7,589 patients). Other countries contributed substantially less. The United States and Canada together contributed only a tenth of the total number of trials contributed by European countries.

**Interpretation:**

Global contributions to randomized trials and the total number of patients recruited have been led by Scandinavian countries and the UK. Countries with few trials but a large burden of hip fractures have an opportunity to engage in high-quality research to resolve important surgical questions and improve the generalizability of study results.

Hip fractures are a significant orthopedic issue because of increasing incidence and associated morbidity and mortality. The total worldwide hip fracture incidence was estimated to be 1.6 million in 2000, and previous projections have estimated that incidence will almost double to 2.6 million in the year 2025 (Cooper et al. 1992). While annual decreases in hip fracture incidence have been noted both in the USA (2.5%) and Canada (1.6%), there is potential for progress given the lower incidences in other countries (Cooper et al. 2011, Dhanwal et al 2011). There is a need for further hip fracture research, and the high incidence of hip fractures also presents a great opportunity for enrollment into clinical trials. However, certain countries such as the Scandinavian nations have published a greater proportion of randomized controlled trials on hip fracture than other countries. If there are indeed discrepancies between certain countries’ published hip fracture trials, there may be pools of untapped research potential. The main purpose of this study was to characterize the patterns of geographic distribution of randomized controlled trials regarding hip fracture surgery. We wanted to identify countries that are proficient in clinical research, as well as countries that have potential for increased contributions and collaboration in clinical trials. Our primary hypothesis was that there are discrepancies globally in national contributions to high-level surgical hip fracture trials.

## Methods

### Eligibility criteria

To be included in the study, a trial had to meet the following criteria: (1) randomized controlled trial; (2) investigated the surgical management of any femoral neck fracture, intertrochanteric fracture, or subtrochanteric hip fracture; (3) included comparison of surgical intervention including implant type, cement use, bone graft/substitute, surgical incision or technique, and implant-guiding technology, but excluded comparisons of rehabilitation, nutrition, medications, and anesthetic interventions; (4) was published between May 1970 and May 2011. Results from all journals in any language were included.

One of the investigators (MY) completed the search independently. The titles and abstracts were screened for adherence to our outlined eligibility criteria. All articles that met the criteria in the initial screen, and any articles that the investigator felt uncertain whether the eligibility criteria were met, were retrieved and scrutinized in a full-text review. Any further uncertainties regarding eligibility criteria were discussed with the second investigator.

### Identification of eligible trials

We searched several electronic databases systematically to identify randomized control trials published between May 1970 and May 2011. The electronic databases searched (through OvidSP) included: EMBASE (from May 1970 to May 2011) and MEDLINE (from May 1970 to May 2011) (OvidSP database, 2011). Regarding search terms, we conducted a keyword search to identify publications matching (1) “fracture$” AND (2) “hip$” or “femur$” or “femoral$” or “trochant$” or “pertrochant$” or “intertrochant$” or “subtrochant$” or “intracapsular$” or “extracapsular$” or “femoral neck” or “femoral head” or “subcapital” or “basicervical” or “transcervical”, and the results were further limited to randomized control trials. The results were uploaded to a bibliographical management database.

### Data extraction

One of the authors (MY) completed a review of all the articles that were identified as meeting our eligibility criteria. The relevant data were extracted from each study, including information regarding the study size and location, population, intervention, and outcomes. The location of the study (including continent, country, city, and specific centers) was extracted from the article whenever possible. If the geographic location of the trial was not explicitly mentioned, the academic address of the first author was used. In the case of multicenter trials, all centers were documented, but the center that was listed first was used as the primary location. Each trial was documented as either a single-center trial or a multicenter trial whenever such information was divulged. The sample size of each study was extracted at the review of the article, using the number randomized for inclusion in the study whenever such a number was specified. The surgical interventions compared in each study were extracted, and the comparison of interventions was classified according to the following. Comparisons of implants or fixation techniques were classified as ‘comparison of implants‘, comparison of the cements, bone grafts were classified as ‘implant modification agents‘, comparisons of implant-related techniques such as reaming and targeting techniques were classified as ‘implant-related techniques’, and studies involving comparison of surgical techniques, approaches, draping, drain usage were classified under ‘general surgical techniques’. Fractures of the femoral neck or those specified by the study as femoral neck fractures were classified as ‘cervical’ fractures, fractures occurring between the greater and lesser trochanters or otherwise specified as intertrochanteric fractures were classified as ‘intertrochanteric’ fractures, fractures occurring below the lesser trochanter to 5 cm distal to the lesser trochanter or otherwise specified as subtrochanteric fractures were classified as ‘subtrochanteric’ fractures, and studies involving an assortment of the above categories or where the specific pattern of hip fracture was difficult to ascertain were classified as ‘mixed’. Furthermore, we recorded publication information for each study, such as date of publication and journal.

### Data and map analyses

Cumulative sums of the numbers of publications on randomized controlled trials and total sample population studied were computed by country and city.

The computer-generated maps were created using Tableau visual data analysis software (Seattle, WA). The cumulative sample sizes studied per city were imported into the mapping software. The software graphically represented the value as a circle at the geographical location of the city, with the area of the circle proportional to the numerical value of cumulative sample size at that city. The values of the enrollment ratio were similarly imported into the mapping software. The software assigned each country a color on a scale from light to dark proportionally representing the above values, with darker colors indicating greater values.

## Results

### Literature search

**Figure 1.**
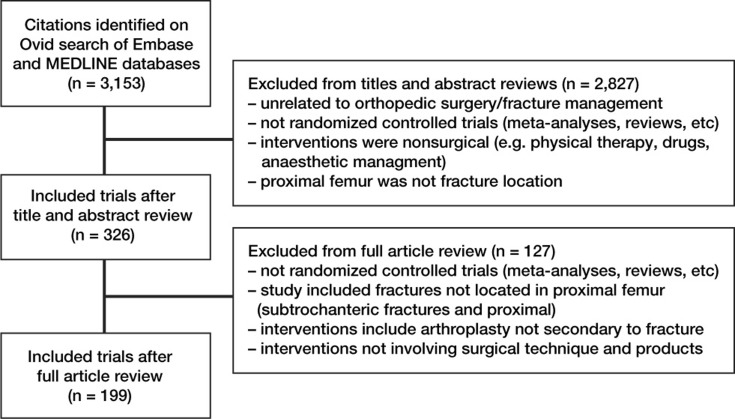
Summary of the literature search and inclusion/exclusion process.

The systematic search using Ovid in both the EMBASE and MEDLINE databases identified 3,153 citations. Initial review of titles and abstracts allowed us to remove of 2,837 studies. Final review of the remaning 326 full articles led to removal of another 127 articles. The reasons for removal are given in Figure 1. Following full review, 199 articles met our inclusion criteria and were included in the analysis (Figure 1).

### Characteristics of eligible trials

The 199 randomized, controlled surgical trials on hip fracture covered 29,119 hip fractures. The majority of these studies were quite recent, with half of them published from 2000 to the present, and one third between 1990 and 1999 (Table 1). Four-fifths of the surgical interventions studied compared implants, though some studies investigated implant modifiers, placement techniques, and general surgical techniques. Single-center trials vastly outnumbered multicenter trials. Of the 199 trials, 24 were multicenter trials.

**Table 1.** Summary of study characteristics of surgical hip fracture trials in our systematic review

Characteristic	No. of studies (% of studies)
No. of articles	199
Total sample size	29,119
Date of Publication	
2010 to present	17 (9%)
2000–2009	89 (44%)
1990–1999	63 (32%)
1980–1989	27 (14%)
1970–1979	3 (2%)
Journal of publication	
J Bone Joint Surg Br	37 (19%)
Acta Orthop	27 (14%)
Injury	22 (11%)
J Orthop Trauma	13 (7%)
Clin Orthop	11 (6%)
J Bone Joint Surg Am	10 (5%)
Arch Orthop Trauma Surg	10 (5%)
Int Orthop	9 (5%)
Ann Chir Gynaecol	5 (3%)
Other	55 (28%)
Type of fracture	
Cervical	93 (47%)
Intertrochanteric	85 (43%)
Subtrochanteric	3 (2%)
Mixed	15 (8%)
Type of trial	
Single-center	172 (86%)
Multicenter	24 (12%)
Type of Intervention	
Comparison of implants	163 (82%)
Implant-modifying agent (cement, grafts)	16 (8%)
Implant placement-related technique (reaming, targeting)	9 (5%)
General surgical techniques	11 (6%)

### Geographic distribution of surgical trials

**Figure 2.**
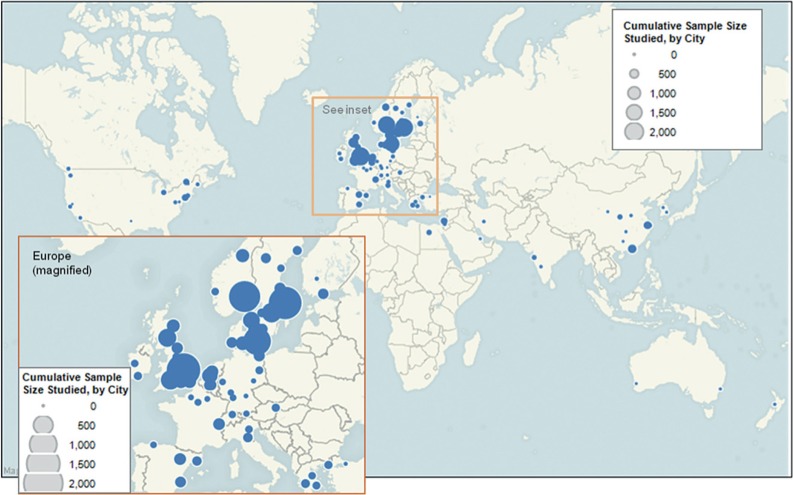
World map showing the cumulative sample sizes of randomized controlled trials on hip fracture, by city.

Europe, with most of the contributions occurring in Scandinavia and the United Kingdom, led in both the number of randomized controlled trials (n = 156) and combined study size (25,388 patients) (Table 2 and Figure 2). North America published 10 times fewer trials (15 trials with a combined sample size of 1,183 patients). The largest cumulative study sample sizes on a national level could be attributed to the multitude of clinical trials conducted in Sweden, the United Kingdom, and Norway. These same countries were also responsible for conducting the most multicenter trials (Table 3).

**Table 2.** The number of randomized controlled trials and combined sample size on a national basis

Geographic region	No. of randomized controlled trials		Total study size	
Oceania	3	1.5%	181	0.6%
Australia	2	1.0%	112	0.4%
New Zealand	1	0.5%	69	0.2%
Europe	156	78%	25,388	87%
Austria	1	0.5%	120	0.4%
Belgium	5	2.5%	462	1.6%
Denmark	8	4.0%	722	2.5%
Finland	4	2.0%	229	0.8%
France	3	1.5%	206	0.7%
Germany	10	5.0%	829	2.9%
Greece	4	2.0%	473	1.6%
Ireland	2	1.0%	222	0.8%
Italy	3	1.5%	266	0.9%
Netherlands	5	2.5%	1,157	4.0%
Norway	13	6.5%	3,022	10%
Spain	5	2.5%	879	3.0%
Sweden	50	25%	8,941	31%
Switzerland	3	1.5%	271	0.9%
United Kingdom	40	20%	7,589	26%
Asia	25	13%	2,367	8.1%
China	11	5.5%	1,247	4.3%
India	4	2.0%	381	1.3%
Iran	1	0.5%	80	0.3%
Israel	3	1.5%	365	1.3%
Nepal	1	0.5%	60	0.2%
South Korea	3	1.5%	138	0.5%
Taiwan	1	0.5%	66	0.2%
Turkey	1	0.5%	30	0.1%
North America	15	7.5%	1,183	4.1%
United States of America	11	5.5%	850	2.9%
Canada	4	2.0%	333	1.1%
Total	199		29,119	

**Table 3.** Geographic distribution of multicenter trials

Country	No. of multicenter RCTs	Total sample size
Sweden	8	2,544
United Kingdom	6	1,161
The Netherlands	2	705
Norway	2	892
USA	2	175
Australia	1	60
Finland	1	426 **^a^**
India	1	102
Spain	1	183
Taiwan	1	66

**^a ^**This study was from a multinational study involving Sweden and Finland; the publication and sample size numbers for both countries were included.

## Discussion

We found that the European nations such as Sweden, Norway, and the United Kingdom contributed the most published randomized, controlled surgical trials on hip fracture and also studied the greatest number of samples. United States and Canada, together with Asian and Oceanic countries, provided considerable contributions but fell behind their European counterparts.

Our study was strengthened by a comprehensive search strategy using MEDLINE and EMBASE databases, but it had certain limitations. Firstly, although publications in all languages were included, the searches used to locate these studies in the various databases were conducted with English search terms. Many articles published in other languages were still identified through this search, but this was probably dependent on the presence of accurate MeSH subject headings and a translated English-language abstract. Thus, it is possible that studies without the benefit of a translated abstract or appropriate tagged English subject headings were not found.

We evaluated hip fracture clinical trial productivity globally. Previous studies have examined international contributions to publications in surgery and orthopedics (Bosker and Verheyen 2006, van Rossum et al. 2007). Another study on trends in randomized control trials in orthopedic surgery in terms of fracture type indeed supported our hypothesis that a discrepancy exists in the global contributions to clinical evidence on hip fracture surgery (Robert et al. 2001). We found that in comparing the number of studies published and cumulative sample size, Scandinavian nations such as Sweden and Norway, and also the United Kingdom, were far more proficient in recruiting patients and conducting high-level evidence hip fracture trials than other nations such as Canada and the United States. It is also important to note the lack of contributions from South American countries and from the African continent in our study. This may be explained by the fact that the rates of hip fracture incidence are lowest in Latin America and Africa (Dhanwal et al. 2011). Similarly, our search did not identify any studies from other countries with intermediate rates of hip fracture, such as Venezuela (Morosano et al. 2005), and Singapore (Koh et al. 2001)—which has the highest hip fracture incidence in Asia (Dhanwal et al. 2011). We believe that the involvement of countries such as these in clinical trials will be crucial since hip fractures continue to be a significant morbidity and mortality issue worldwide. In particular, it has been predicted that Asia will be the source of over half of the world’s total of osteoporotic fractures by 2050 (Cooper et al. 2011). These nations, which have so far contributed little to surgical trials on hip fracture, have an opportunity to use their pool of fractures to answer important surgical questions.

There are probably many other factors at play to explain the geographic discrepancy in hip fracture trial contributions. Previous studies have identified various other factors affecting output of surgical publications, such as proficiency in the English language, national research funding (Man et al. 2004), and population size (van Rossum et al. 2007). In terms of research and development expenditure as a percentage of GDP, Sweden, Finland, Denmark, and Norway rank 2, 3, 9, and 17 in the world out of 34 nations measured (Organization for Economic Co-operation and Development 2008). Other key contributors in hip fracture research identified in this study also ranked high in research spending as a percentage of GDP: the United States (7), Germany (8), and the United Kingdom (14). Population size does not appear to have been an important factor in our study, as most contributions came from medium-sized populations.

In addition, high-contributing countries such as the United Kingdom, Sweden, and Norway have national healthcare systems with resources such as national hip fracture registries to facilitate data collection—the Norwegian Hip Fracture Register (Gjertsen et al. 2008), the National Hip Fracture Database in the United Kingdom (Currie et al. 2011), and the Swedish RIKSHÖFT-SAHFE (Thorngren and Hommel 2008). Scandinavian countries also have a unique personal identification number for all residents, which allows ease of access to healthcare information for clinical investigations. Many countries, including those in North America, lack this sort of national resource for accessing data and follow-up outcomes, which may have contributed to the lower contributions seen in the present study. These differences in funding, national healthcare resources and registries, and national regulations and standards can also explain the disparities in surgical trial output within Europe.

Barriers to conducting surgical clinical trials such as limited training in research methodology, patient preference, and lack of clinical equipoise have been discussed thoroughly in the literature, although the amount of international variation is unknown (Bedermen et al. 2010). However, issues such as funding and the influence of healthcare systems may present unique challenges in certain countries. The presence of regulatory boards for surgical interventions in certain countries can motivate research. In the United Kingdom, which was proficient in conducting surgical hip fracture trials, the National Institute for Health and Clinical Excellence regulates the introduction of new surgical interventions (Organization for Economic Co-operation and Development 2008). These regulatory standards provide incentive for researchers to conduct trials to provide evidence for surgical procedures. In Canada and the United States, where no such regulatory body for surgical procedures exists, there is less driving force to pursue scientific evaluation through clinical trials. Orthopedic clinical trials present unique challenges that require an adequate research infrastructure and experienced investigators for success (Trippel et al. 2007). The complicated infrastructure required for a successful trial not only involves the the principal investigator but may also involve data coordination centers, steering committees, adjudication committees, data safety monitoring boards, etc. (Wright et al. 2011). This might explain the geographic patterns we see in this study: nations proficient in research continue to conduct large studies, while those without such established infrastructure and experience do not have this output. Although it is difficult to measure quantitatively, a strong culture of research in these European nations may be a factor in their strong contributions to hip fracture research; in a recent study, Sweden, Finland, Norway, and Denmark ranked 1, 3, 4, and 5 respectively, in population-corrected rates of orthopedic publication (Bosker and Verheyen 2006). There is need for collaboration in this regard, as surgeons experienced in research should have the opportunity to provide their expertise in assisting and facilitating national studies in other regions and eventually involve these nations in multinational trials.

Funding has also proven difficult to obtain for orthopedic trials, as peer-reviewed and national funding for clinical trials is limited and insufficient to cover the multi-million dollar costs of well-conducted trials (Bhandari et al. 2009). Certain nations may face unique funding difficulties: for example, in the United States the proportion of the National Institutes of Health budget used for funding of musculoskeletal research is decreasing and is not keeping stride with research opportunities (Haralson and Zuckerman 2009). Furthermore, the absence of regulatory boards may further affect funding in different countries, as the implant industry may not be as willing to provide financial support without the demand for rigorous scientific study prior to surgical product release (McLeod 1999).

An important issue to keep in mind when assessing the significance of the geographic contributions regarding hip fracture trials is the external validity and generalizability of the results on a worldwide basis. While there has been little research on the external validity of surgical or orthopedic trials when applied to different geographic regions, there are certainly factors that would influence the generalizability of study results. The setting of a trial has often been cited as affecting external validity, due to factors such as differences in healthcare systems (Rothwell 2005, Boutron et al. 2008). The European Carotid Surgery Trial is a commonly cited study that illustrates the effect on patient outcomes of different healthcare systems and the relative speed of patient investigation (Rothwell 2005, Boutron et al. 2008). Similarly, variations in healthcare systems such as differences in access to care and timing of surgery may affect the hip fracture outcomes, and thus their generalizability across regions. National differences in societal and cultural behavior, ethnic variances in osteoporosis, and other factors not easily described in the methodology of a clinical trial may influence study outcomes. With such considerations in mind, one can see the importance of involving more countries in hip fracture trials as hip fracture research evolves.

Our findings support our hypothesis and highlight a disparity in geographic contributions to orthopedic hip fracture trials worldwide. The major message of this study is that there is a real opportunity to increase recruitment of hip fracture patients into randomized clinical trials, particularly in those countries with lower contributions. Such opportunities can be used not only by conducting locally-based trials, but also through international collaboration in large multinational trials. Improving contributions worldwide would increase the total amount of evidence available to answer important orthopedic questions, and would enhance the external validity of the hip fracture literature and provide a more global viewpoint.
